# Social interactions do not affect mycoplasma infection in griffon vultures

**DOI:** 10.1098/rsos.240500

**Published:** 2024-12-11

**Authors:** Elvira D'Bastiani, Nili Anglister, Inna Lysnyansky, Inna Mikula, Marta Acácio, Gideon Vaadia, Kaija Gahm, Orr Spiegel, Noa Pinter-Wollman

**Affiliations:** ^1^ Department of Ecology and Evolutionary Biology, University of California Los Angeles, Los Angeles, CA, USA; ^2^ School of Zoology, Faculty of Life Sciences, Tel Aviv University, Tel Aviv, Israel; ^3^ Mycoplasma unit, Department of Avian Diseases, Kimron Veterinary Institute (KVI), Beit Dagan, Israel

**Keywords:** feeding, infectious disease, movement ecology, pathogen transmission, roosting, social interactions

## Abstract

Uncovering the ways in which pathogens spread has important implications for population health and management. Pathogen transmission is influenced by various factors, including patterns of social interactions and shared use of space. We aim to understand how the social behaviour of griffon vultures (*Gyps fulvus*), a species of conservation interest, influences the presence or absence of mycoplasma, a group of bacteria known to cause respiratory diseases in birds. We investigated how direct and indirect social interactions of griffon vultures in the wild, in different social situations, impacted the mycoplasma infection status. We inferred interactions from high-resolution global positioning system (GPS) tracking data. Specifically, we assessed how social behaviour affects infection status when vultures share feeding and roosting locations, either at the same time (direct interactions) or subsequently, when space use is asynchronous (indirect interactions). We did not detect a significant effect of any social situation and type of interaction on infection status. However, we observed a high population prevalence of mycoplasma, suggesting that other factors might be more important than social interactions in determining the transmission of this bacteria in the Israeli vulture population. Uncovering the mechanisms that underlie infection status in wildlife is crucial for maintaining viable populations, designing containment management actions and gaining insights into the ecological mechanisms that drive infectious disease dynamics.

## 1. Introduction

Uncovering the ways in which pathogens spread through a population is crucial for mitigating the transmission of pathogens, with implications for population health and management. Pathogen transmission is influenced by many factors including the transmission route, which may be facilitated by direct and/or indirect interactions among potential hosts [1,2]. Traditional epidemiological studies utilize theoretical models and social network analysis to investigate pathogen spread [1,3–6]. While these studies can explicitly consider how host interactions mediate pathogen transmission, empirical studies testing these questions *in situ* are challenging because of the costs incurred by investigating pathogen spread throughout an entire population [3,6–11]. Understanding what factors influence exposure to pathogens including social behaviour and host attributes, is crucial for enhancing wildlife conservation efforts. Despite extensive investigations into pathogen spread and the development of sophisticated host–pathogen models, our understanding of the factors influencing infectious disease prevalence in wild animal populations remains limited.

These questions are particularly difficult to disentangle since transmission of pathogens is affected by the characteristics and biology of each infectious agent. Pathogens differ in their transmission modes (airborne, waterborne, vector-borne, food-borne, faecal-oral, etc.); therefore, it is important to investigate different social and ecological situations that may facilitate pathogen transmission, as well as the environment. For example, airborne pathogens such as *Mycoplasma gallisepticum*, which infects birds, can be transmitted through airborne droplets when individuals are in close physical proximity and share airspace. In contrast, non-airborne pathogen transmission might require the sharing of a feeding site or drinking water contaminated with infectious agents [12–14]. Thus, exploring pathogen spread is important for developing specific strategies to manage infectious disease dynamics in wild populations, such as periodic vaccination programmes or interventions to reduce the risk of pathogen transmission at specific locations.

It is important to determine which attributes contribute to pathogen acquisition and spread to inform effective disease management. Pathogen transmission can be influenced by host susceptibility and the host’s contact or exposure to the pathogen [15–17]. Individuals often have different social roles in a population, which may impact how pathogens spread [8,18–22]. For example, individuals that contact many others are more prone to infection [11]. Similarly, individuals with more unique social partners are more likely to become infected with an infectious disease that is transmitted through social interactions due to increased exposure to infected individuals and their pathogens [8,11]. Furthermore, individuals who interact frequently with others might be more prone to infectious diseases that are transmitted through multiple exposures to a pathogen [5,23]. For instance, Japanese macaques (*Macaca fuscata fuscata* and *M. fuscata yakui*) that engage more frequently in grooming interactions are more likely to become infected with nematodes [24]. In addition to social roles, host attributes such as age can impact infectious status, for example because of changes to the immune system as animals age that might alter susceptibility [15,25–27]. For instance, in house finches and raptors, the prevalence of mycoplasma is higher in juveniles than in adults [25,28,29]. Uncovering how host attributes affect infectious disease dynamics can provide important information for managing the spread of pathogens, for example, by recommending the removal or vaccination of certain individuals that have potentially high impact on pathogen transmission [5,15,25,30]. Such understanding is also important for gaining knowledge about the ecological elements that drive the persistence of infectious diseases.

Griffon vultures (*Gyps fulvus*) [31] are social scavengers that interact when feeding and roosting and are exposed to a wide range of pathogens. The study population in Israel is locally critically endangered [32,33] and has been the target of many conservation efforts including the deployment of global positioning system (GPS) tags on the majority of the population. Because population size is a concern in the region, it is important to understand the potential causes of population decline including infectious disease dynamics. In the griffon population that we studied, mycoplasma has very high prevalence and more than one strain has been identified, as detailed by Anglister *et al*. [29]. Mycoplasma can cause a reduction in the vultures’ flight distances, particularly in sub-adults, potentially reducing their ability to find food [29]. Despite its prevalence and impacts on griffon behaviour, we know very little about how this bacteria spreads in the population. Griffon vultures aggregate at communal roosts and around carcasses [34]. They use their night roosts to share information about the location of feeding sites [35], where they often feed together, exchanging bodily fluids through regurgitations. Griffon vultures differ in their social position across social situations [36]; therefore each individual may have a different impact on disease spread dynamics. Because pathogens can persist in the environment, shared spaces such as communal roosts or feeding sites are potential sources for indirect pathogen transmission. The extent to which shared space use contributes to pathogen transmission and spread depends on the specific characteristics and biology of a pathogen.

Mycoplasma belongs to the class Mollicutes, which lacks a cell wall [37]. The transmission of mycoplasmas depends on the species and can be horizontal, through contact with infected individuals, contaminated surfaces, or airborne particles and/or vertical, from an infected mother to her offspring [38–42]. Mycoplasma can persist in the environment for days, weeks, or even months [43–46]. More than 20 mycoplasma species have been found to infect birds [46,47], including more than one strain in griffon vultures [29,48]. Nevertheless, due to the genetic differences among mycoplasma species, their impact on hosts may vary [49–51]. Some mycoplasma species are commensals while others are pathogenic and their impact on the host will depend on the host body condition and presence of other pathogens [47,52–55]. Pathogenic mycoplasma species can cause acute or chronic conditions including respiratory infections, conjunctivitis, arthritis, embryonic death, skeletal deformations and reduced hatchling sizes, depending on the host species and the individuals they infect [28,37,49,53,56–63]. Accordingly, high prevalence of mycoplasma often reduces host survival in the wild [41,49,64]. However, the effects of mycoplasma in non-passerines remain poorly understood [28,49], despite the high prevalence of the bacterium in some populations.

**Figure 1.**
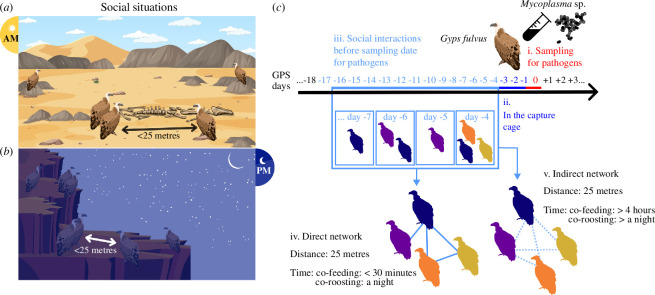
Constructing social networks to investigate the impact of social interactions when vultures are feeding (*a*) or roosting (*b*) on infection status (positive or negative) with mycoplasma. The timeline (*c*) illustrates when social interactions are considered before sampling for pathogens: (*i*) day on which vultures are sampled for pathogens; (ii) days when the vultures were in the capture cage (excluded from social interaction analysis); (iii) days used to examine social interactions; (iv) direct interactions occur within 30 min for co-feeding or over one night for co-roosting; (*v*) indirect interactions were recorded when more than 4 h, for co-feeding, and more than one night, for co-roosting, elapsed between observations of vultures within 25 metres of each other.

Here, we investigate how the social behaviour of wild griffon vultures relates to infection with mycoplasma. We examine how direct and indirect social interactions in different social situations (feeding and roosting), relate to mycoplasma infection status in a wild vulture population (figure 1). We predicted that social interactions while feeding would have a greater impact on infection status than interactions while roosting because during feeding, individuals might share bodily fluids (mainly aerosols) due to food sharing and regurgitation, while during roosting, interactions might be less intense. Alternatively, interactions while roosting might be a better predictor of infection status compared with feeding interactions, because vultures spend more time with one another overnight at the roost, resulting in potentially longer exposures to mycoplasma. Furthermore, we predicted that direct social interactions would have a greater impact on infection status than indirect interactions, because direct contact between individuals may increase the likelihood of pathogen transmission through physical contact or exchange of bodily fluids. In contrast, indirect shared space use may involve contact only through the shared environment, reducing the chance of transmission due to factors such as environmental dilution and shorter exposure durations [65].

## 2. Methods

### 2.1. Study system

The Eurasian griffon vulture is a social scavenger that engages in frequent social interactions when feeding, roosting, resting and flying. Over the past two decades, the species has experienced a rapid population decline in Israel, from over 500 to fewer than 180 individuals [66]. To combat the population decline, the Israel Nature and Parks Authority (INPA) maintains a management programme that includes food provisioning at feeding stations (e.g. goats or cow carcasses), annual population counts, captures, tracking of individuals and pathogen sampling. In September–November, when vultures are not breeding, they are captured in cages baited with large mammal carcasses every one–three weeks, resulting in the capture of approximately 100 unique griffons yearly, as well as many recaptures.

Among the captured individuals, a total of 114 vultures (87 individuals in 2021 and 93 in 2022) were fitted with GPS-GSM-Accelerometer tags (Ornitrack—50 3G transmitters) using a Teflon harness in a leg-loop configuration (for more details see [67,68]). The GPS tags provide information on vulture locations approximately every 10 min during the day. Vultures are active during the day and, to preserve battery, the solar-powered GPS tags operate only during daylight hours, providing one or two locations at night (for more details see [36]). The high spatial and temporal resolution of the GPS information allows us to infer social interactions in different social situations based on temporal and spatial proximity [36] (for more details see the ‘Script S1a-b’ in electronic supplementary material, ‘code section’). During captures, individuals were inspected for injuries or clinical signs of disease and sampled for pathogens. A total of 77 unique vultures with active GPS tags were examined. Individuals are often recaptured, but are usually not sampled again for mycoplasma to minimize stress. If an individual was captured and sampled multiple times, we only used information from the first sample collected to infer infection status. Vulture age is determined based on the moulting of the primary and secondary flight feathers as well as the eye and ruff plumage colours [69,70]. Individuals aged 0 to 4 years, characterized by a dark bill, dark eye, ruff with lanceolated feathers, and pointy dark reddish-tawny contour feathers are classified as immature; individuals aged more than 4 years have changes that advance with ageing such as lighter cream-coloured bill, brownish to yellow clear eyes, beige to white downy ruff, rounded contour feathers and are categorized as adults [69,70]. Thanks to the long-term capture and monitoring effort by the INPA many of the vultures included in this study were previously trapped as immatures, facilitating accurate ageing of adults.

### 2.2. Characterizing social networks from spatial and temporal data

We examined interactions only of vultures that had been GPS-tracked during the 14 days prior to sampling for mycoplasma. We included only individuals who stayed within the local geographic region of southern Israel, specifically within a 400 km radius of their tagging location. After applying these temporal and geographic filters, we retained high-quality ecological movement data for 114 vultures, representing at least 65% of Israel’s vulture population and nearly all griffon vultures in the south of the country. Simulation studies show that tracking 20% of the effective population provides approximately 75% accuracy of network measures [71], thus, our data probably provided very high accuracy for the network measures we quantified. We excluded from the social interaction analysis the 3 days during which the vultures were in the capture cage (figure 1*c*) to account for any potential influence (e.g. social interactions imposed by cage confinement and their impact on mycoplasma transmission inside the cage) on our results. Our analysis focused on interactions that occurred during the 14 days preceding pathogen sampling and cage confinement because the incubation period of mycoplasma can range from 2 to 23 days. We took 14 days as a midpoint of this range and show in the electronic supplementary material that our results are not sensitive to using slightly longer or shorter periods (electronic supplementary material, tables S3 and S4 and figures S1–S4). Seven sampling events were included in our analysis and we constructed different interaction networks for each sampling event (see table 1 and electronic supplementary material, table S1 for information on each of these networks).

**Table 1.** Sampling date, social network size (i.e. the number of the griffon vultures tracked within a 14-day period leading up to pathogen sampling), and prevalence of mycoplasma at the genus level on each sampling day. Note that individuals in the social networks were not captured on the sampling date, but rather were tagged at previous captures. Furthermore, the number of individuals sampled for mycoplasma on each sampling date only includes individuals that were captured on the sampling day, already had a GPS tag on them, and were not sampled previously for mycoplasma, as detailed in the text.

sampling date	social network size				individuals sampled for mycoplasma			
	direct interactions		indirect interactions		number sampled	negative	positive	prevalence
	feeding	roosting	feeding	roosting				
1 (2021−09−13)	27	28	27	27	2	0	2	100%
2 (2021−09−29)	46	48	39	49	2	0	2	100%
3 (2021−10−07)	60	71	46	65	24	4	20	83%
4 (2021−10−22)	67	69	69	70	2	0	2	100%
5 (2021−11−09)	58	58	42	59	17	4	13	76%
6 (2022−10−03)	66	79	58	79	5	0	5	100%
7 (2022−11−03)	70	79	69	80	24	12	12	50%
**average:**	56.285	61.714	50	61.285	10.85	2.85	8	—

We constructed social networks for two social situations: feeding and roosting (figure 1*a*,*b*). An interaction was inferred when two vultures were within 25 m of one another, when not flying (i.e. moving at a speed of less than 5 m s^−1^), during the day for feeding interactions (figure 1*a*) and during the night for roosting interactions (figure 1*b*). We used a 25 m distance threshold based on biological considerations of mycoplasma [72] and vulture behaviour, and we show in the electronic supplementary material that our results are not affected by using slightly different distance thresholds (electronic supplementary material, tables S5 and S6 and figures S5–S8). Roosting interactions were only considered if they occurred within a known roost site, during the night, as defined in Sharma *et al*. [36]. For feeding interactions, we excluded daytime interactions that occurred within known roost sites.

To distinguish between direct and indirect interactions, we used different time thresholds (figure 1*a*–*c*). We considered direct co-feeding interactions if vultures were feeding within 25 m of each other within 0–30 min, and considered indirect co-feeding interactions if vultures were feeding within 14 days but at least 4 h apart (figure 1*a*). Because vultures may stay near a feeding station for a long period (up to 4 h), if vultures were within 25 m of each other within 30 min and 4 h, we did not consider those interactions to ensure that there is no ambiguity between direct and indirect co-feeding interactions. A 30 min time threshold for data that is collected every 10 min is a very conservative time window that still allows detecting direct interactions. Furthermore, because vultures stay at a carcass for hours, and when they arrive, they approach it slowly, not considering co-locations that occur within 31 min to 4 h, avoids misclassifying as an interaction the co-location of an individual that recently left and one that just arrived at a carcass. Similarly, direct co-roosting interactions were recorded if vultures roosted within 25 m (distance threshold) of each other on the same night. Indirect co-roosting interactions were recorded if vultures roosted within 25 m of each other more than one night apart but less than 14 nights apart (figure 1*b*). To quantify the edge weight between pairs of vultures (strength), we used the number of occasions on which two vultures were observed together.

To examine interactions in both social situations together (co-feeding and co-roosting combined) we created an aggregate network [73]. The weight of each interaction in the aggregate network was the sum of the weights of interactions in the co-feeding and co-roosting situations. For example, consider two vultures, *i* and *j*, with an edge weight of 2 when co-feeding and an edge weight of 3 when co-roosting. In the aggregate network, the edge connecting *i* and *j* would have a weight of 5, representing the cumulative interactions when both feeding and roosting.

### 2.3. Quantifying social role of individuals

To determine the social position of individuals within the social network, we used individual-level centrality measures [74,75]. We used *betweenness* to quantify the extent to which a vulture serves as a bridge or intermediary between other individuals [76]. An individual with high betweenness is likely to facilitate the rapid spread of a pathogen [77,78]. We used *degree* to quantify the number of unique individuals that a vulture interacted with [79]. A vulture with high degree is exposed to more individuals and their pathogens. We used *strength* to describe the frequency of interactions of each vulture [23]. An individual with high strength has more social interactions and, therefore, potentially more pathogen exposure opportunities. To account for different network sizes in the seven different sampling days, we normalized the centrality measures by using the ‘normalize’ argument for betweenness and degree in the respective functions in ‘*igraph*’. This normalization divides degree or betweenness by the number of individuals in the network minus one. To normalize strength, we divided individual strength by the total strength of all edge weights in each network. Network analysis was conducted using the ‘*igraph*’ R package [80].

### 2.4. Mycoplasma data

We sampled 77 unique griffon vultures (out of the 114 GPS-tracked individuals used to analyse social interactions) for the presence and absence of mycoplasma (table 1 and electronic supplementary material, tables S1 and S2). We collected samples from the vultures’ choanal or tracheal mucosa using a sterile swab and stored them at −20°C until DNA extraction. The DNA was extracted directly from individual choanal/tracheal swabs by agitating them vigorously in 1 ml of phosphate-buffered saline (PBS; Sigma, Rehovot, Israel). Genomic DNA was then extracted from 400 µl of PBS solution using the Maxwell DNA Isolation Kit for Cell/Tissue and the Maxwell® 16 apparatus (Promega), following the manufacturer’s instructions.

The extracted DNA was amplified using the forward GPF primer (5' GCT GGC TGT GTG CCT AAT ACA 3' [58]; and the reverse MGSO primer (5' TGC ACC ATC TGT CAC TCT GTT AAC CTC 3' [81]. The polymerase chain reaction (PCR) was based on the 16S rRNA gene (approx. 1000 bp in length), and reactions were performed in 25 µl volumes, consisting of 0.5 µl of Phire Hot Start II DNA Polymerase (Thermo Fisher Scientific, Waltham, MA, USA), ×5 Phire reaction buffer, 1 µl of 10 mM dNTPs, 0.4 µM of each primer and 5 µl of DNA. The PCR amplifications were carried out using a C1000 Touch™ Thermal Cycler (Bio-Rad, Hercules, CA, USA). The amplification procedure was conducted as outlined by Lierz *et al*. [58] with a slight modification: initiating incubation at 94°C for 3 min, followed by 35 cycles of denaturation at 94°C for 30 s, annealing at 66°C for 30 s and synthesis at 72°C for 1 min. The process concluded with a final extension at 72°C for 5 minutes. DNA of *Mycoplasma falconis* was used as a positive control, while nuclease-free water (Sigma, Rehovot, Israel) served as a negative control.

The amplified PCR products were separated in a 1% agarose gel and visualized using ethidium bromide staining and ultraviolet transillumination. A biomarker (bp−100 Bio-Rad, Hercules, CA, USA) was used to determine the size of DNA fragments. The positive PCR samples were purified using the MEGAquick-spin^TM^ PCR & Agarose Gel DNA Extraction System (iNtRON Biotechnology) and if the PCR yielded enough genetic material, the samples was subjected to Sanger sequencing (Hylab Ltd, Rehovot, Israel) using the Applied Biosystems DNA sequencer and the ABI BigDye Terminator cycle sequencing kit (Applied Biosystems, Foster City, CA). The sequence editing, consensus generation, and alignment construction were conducted using Lasergene software (version 5.06/5.51, 2003, DNAStar, Inc., Madison, WI), and Geneious software version R9 (https://www.geneious.com/academic/). Additionally, we compared the nucleotide sequences of the resulting amplicons with data deposited in GenBank (for more details, see [29]). Finally, we measured the prevalence of mycoplasma (genus level) on each sampling date. Mycoplasma prevalence was calculated by dividing the number of individuals infected by the total number of sampled individuals (table 1), and then multiplying the result by 100 to express it as a percentage [82].

### 2.5. Statistical analysis

To determine the relationship between social position and infection status we used generalized linear mixed models (GLMMs) with a binomial distribution of errors [83–85]. We ran a separate model for each type of interaction (co-feeding direct, co-feeding indirect, co-roosting direct, co-roosting indirect, aggregate direct and aggregate indirect) resulting in six statistical models when examining infection with bacteria from the mycoplasma genus. Infection status (yes/no) was the response variable, and the centrality measures betweenness, degree and strength were the fixed effects. We incorporated age (immature/adult) as a fixed effect in the model to account for the impact that age might have on infection status, which has been observed in other studies [29]. Approximately half of the samples were from adults and half were from immature individuals. We included the sampling date as a random effect in all models to account for variation that might be introduced by sampling vultures on different days. We determined if the underlying model assumptions were met by examining collinearity of fixed effects, random effects distribution, homoscedasticity, independence and normality of residuals [83]. Before analyses, we tested all of the variables and did not find collinearity using a variance inflation factor test (VIF < 3). For more details about the GLMM analysis see table 2 and electronic supplementary material, tables S3 and S4. In addition, we applied the Bonferroni correction to the GLMMs models to account for multiple comparisons. To account for multiple comparisons, because we ran six models, we used a *p*-value threshold of 0.0083 (0.05/6) to determine statistical significance, rather than the traditional 0.05 threshold. We conducted all statistical analysis in R version 4.3.1 [86] using the ‘*DHARMa*’ [87], ‘*lmer4*’ [84], ‘*Performance*’ [88] and ‘*Stats*’ [89] packages. Data and analysis code can be found at https://github.com/elviradbastiani/MycoplasmaProject_2023.

**Table 2.** Results of the binomial generalized linear mixed model (GLMM) testing the relationship between mycoplasma infection status and social position (degree, betweenness, and strength) of griffon vultures.

social situation	type of interaction (sample size)	fixed effect	estimate	standard error	*z*-values	*p*‐value
co-feeding	direct (*n* = 68)	intercept	0.582	0.769	0.757	0.449
		degree	0.431	2.047	0.211	0.833
		strength	10.323	26.500	0.390	0.697
		betweenness	−1.150	7.322	−0.157	0.875
		age (immature)	1.047	0.632	1.657	0.097
	indirect (*n* = 63)	intercept	0.530	0.582	0.911	0.362
		degree	0.392	1.670	0.235	0.815
		strength	−4.540	10.910	−0.416	0.677
		betweenness	0.967	4.495	0.215	0.830
		age (immature)	1.057	0.626	1.689	0.091
co-roosting	direct (*n* = 76)	intercept	0.697	0.680	1.025	0.305
		degree	−2.787	5.054	−0.551	0.581
		strength	55.968	59.377	0.943	0.346
		betweenness	−0.105	16.182	−0.007	0.995
		age (immature)	1.193	0.632	1.888	0.059
	indirect (*n* = 75)	intercept	0.844	0.725	1.164	0.245
		degree	−1.068	2.923	−0.365	0.715
		strength	44.370	43.687	1.016	0.310
		betweenness	−9.777	15.671	−0.624	0.533
		age (immature)	1.201	0.641	1.873	0.061
aggregate networks	direct (*n* = 76)	intercept	0.805	0.742	1.085	0.278
		degree	0.050	1.555	0.032	0.974
		strength	6.839	26.177	0.261	0.794
		betweenness	4.200	17.410	0.241	0.809
		age (immature)	1.095	0.614	1.784	0.074
	indirect (*n* = 76)	intercept	1.085	0.873	1.243	0.214
		degree	1.890	1.800	1.050	0.294
		strength	−20.539	17.170	−1.196	0.232
		betweenness	−18.167	18.286	−0.994	0.320
		age (immature)	1.027	0.631	1.627	0.104

## 3. Results

During the two years of the study (2021–2022), there were seven capture events in which vultures were sampled for mycoplasma, resulting in 28 social networks (table 1). In our tracking dataset, based on the criteria we applied, we observed a total of 106 individuals interacting while feeding and 114 individuals interacting while roosting. Of these, 77 unique individuals were sampled for pathogens. We examined the relationship between social behaviour and infection status, considering mycoplasma identification at the genus level.

**Figure 2.**
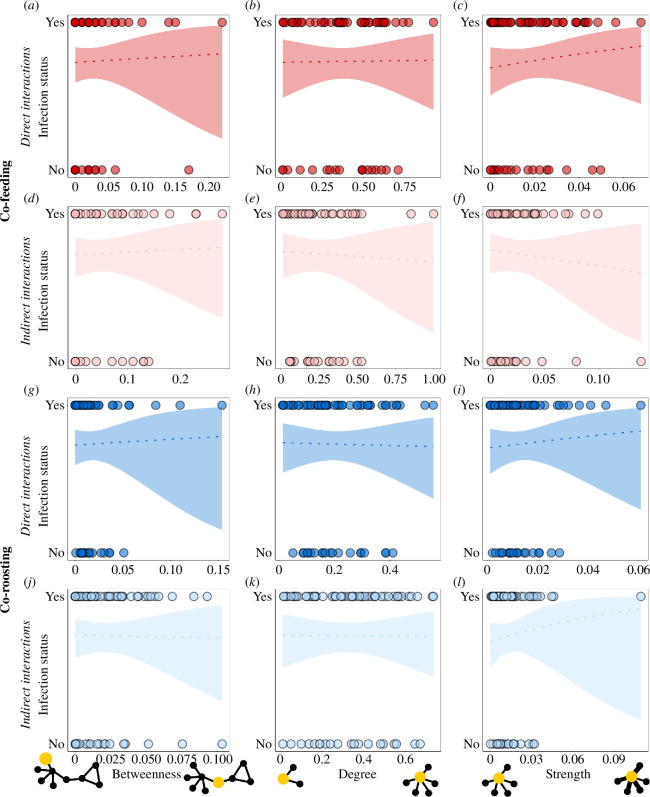
Relationship between social position (betweenness (*a,d,g,j*), degree (*b,e,h,k*), and strength (*c,f,i,l*) of griffon vultures and infection with mycoplasma. We examined both direct (*a–c,g–i*) and indirect (*d–f,j–l*) interactions when vultures were co-feeding (*a–f*) or co-roosting (*g–l*) during the 14 days before they were sampled for mycoplasma. Here and in the following figure, lines are the GLMM fit, shaded areas are the 95% confidence interval and points are the raw data.

**Figure 3.**
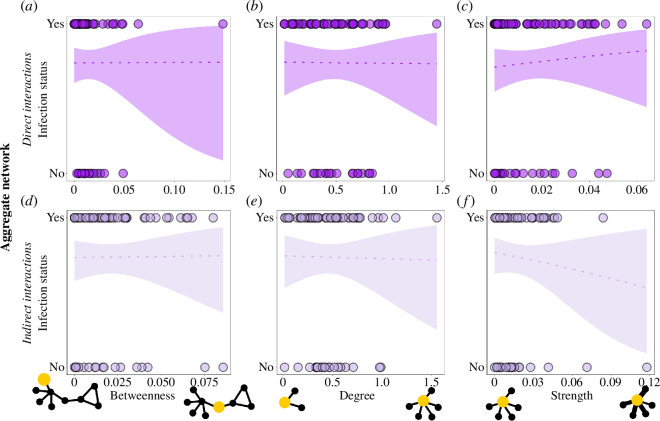
Relationship between social position (betweenness (*a,d*), degree (*b,e*) and strength (*c,f*)) of griffon vultures and infection with mycoplasma. We examined both direct (*a–c*) and indirect (*d–f*) interactions when co-feeding and co-roosting interactions were aggregated into a single network.

In contrast to our expectations, vulture infection with mycoplasma was not related to social position in any type of interaction network (figure 2, table 2). This was the case even after combining direct interactions when feeding and roosting into a single network (figure 3*a*–*c*, table 2), and when combining indirect interactions when feeding and roosting into a single network (figure 3*d*–*f*, table 2). We further did not find a significant relationship between infection status and age, although immature were slightly, but not statistically significantly, more likely to be infected than adults (table 2). To ensure that these results are not biased by days in which sample sizes were smaller, we repeated the analysis using only the three days with the largest sample size (sampling days 3, 5 and 7) and the results were consistent with those obtained from the full dataset (see electronic supplementary material, figure S9 and table S7).

In most statistical models examining infection with the *Mycoplasma* genus, we found that some variation in infection status was attributed to the sampling date. The random effect ‘sampling date’ accounted for 45% (s.d. ± 0.671) of the variance in the model for co-feeding direct interactions. For models of indirect co-feeding interactions, the random effect ‘sampling date’ accounted for 0.01% (s.d. ± < 0.0001) model variance. For models of direct co-roosting interactions, the random effect ‘sampling date’ accounted for 35.9% (s.d. ± 0.599) model variance. For models of indirect co-roosting interactions, the random effect ‘sampling date’ accounted for 47.1% (s.d. ± 0.686) model variance. In the models of the aggregated network (co-feeding + co-roosting) the random effect ‘sampling date’ accounted for 49.1% (s.d. ± 0.701) of the variance in the model for direct interactions, and 99.1% (s.d ± 0.995) of the variance in the model for indirect interactions.

## 4. Discussion

Contrary to our predictions, we found that social behaviour did not affect vultures’ mycoplasma infection status. This finding held regardless of the type of interactions (direct or indirect) or the social context (feeding or roosting). Our inability to detect an effect of social interactions on infection status is probably due to the very high prevalence of mycoplasma in the population [29]. We sampled 77 individuals for mycoplasma, a very large sample size for this kind of study, therefore, our inability to detect an effect is probably not because of low statistical power, but mainly due to the high prevalence of mycoplasma in the population. It is possible that the high prevalence of mycoplasma in the population is influenced by factors we did not examine such as contaminated commonly used food or water sources, climatic conditions, or chronic carrying of the bacteria without pathology, as we discuss in more detail below.

Mycoplasma infection status was not related to social behaviour during feeding or roosting, suggesting that the social interactions of griffon vultures do not impact mycoplasma infection. Furthermore, our findings did not change when we examined longer (21 or 28 days), or shorter (7 days) time periods during which vultures interacted (electronic supplementary material, §4) suggesting that our lack of significant results is not because our sampling period of social interactions was shorter or longer than the potential incubation period of mycoplasma. The interactions we examined are based on a relatively stable population and individuals may appear in more than one interaction network. However, repeated appearances of individuals in different social networks do not necessarily mean that the same interactions occurred in the different periods, and there are no repeated measures of individuals sampled for mycoplasma. Inferring social interactions using GPS data does not necessarily reflect direct contacts between individuals due to positional accuracy and precision errors. Still, inferring interactions based on spatio-temporal co-occurrence is one of the most common approaches to the study of animal social networks, as it provides the benefits of remote sensing that do not impact the observed interactions, which might occur when observations are present [90]. Our findings contrast with previous studies in which birds that were more social were also more likely to be infected with mycoplasma [12,14,91–94]. Thus, social behaviours may have different implications for mycoplasma spread across different bird and bacteria species [49,64]. While Adelman *et al*. [14] found that songbirds feeding with more conspecifics exhibit a higher likelihood of transmitting mycoplasma, we did not observe a relationship between the number of conspecifics with which a vulture feeds and mycoplasma infection. Future work might compare interactions of vultures at feeding stations with interactions at naturally occurring carcasses, because feeding stations might be more contaminated than sites of naturally occurring carcasses. This difference could be explained by behavioural differences among the two host species. Species of birds differ in their social behaviours, immune responses and susceptibility to infections, and may experience different environmental conditions, all of which can influence disease prevalence and transmission. Indeed, other bird species have lower mycoplasma prevalence than the high prevalence we observed here [64]. One way in which griffon vultures are different from songbirds is their robust immune system, which is probably shaped by their scavenging behaviour. The physiological and immunological characteristics of vultures [95] may make them less prone to pathological impacts of mycoplasma, particularly compared with other bird species like songbirds.

There are many possible explanations for the high prevalence of mycoplasma observed in our study. Mycoplasma bacteria can be commensal and/or pathogenic. Mycoplasma can act as a commensal in the respiratory tract without causing diseases, allowing it to persist in the host population without eliciting clinical signs or causing harm [46]. Such persistence in a non-harmful state can lead to chronic infection in which the bacteria is present in a large portion of the population. Indeed, several mycoplasma species associated with respiratory diseases in birds are known to cause chronic conditions [60,96]. Future work on the infection status of individuals that are sampled repeatedly over time is needed to determine whether mycoplasma is a chronic disease in our system as well. Certain species of mycoplasma can be pathogenic, causing respiratory diseases, especially under certain conditions such as compromised host immune system and hot weather [97–100]. However, we did not observe obvious clinical symptoms in our study. It is possible that in our study system some species of mycoplasma are commensal, while others are pathogenic (or become pathogenic at some point). Finally, there is no evidence of co-infection with multiple species of mycoplasma in this system [29], so it is unlikely that there were synergistic effects of different mycoplasma species on the clinical state of the vultures.

Pathogenic bacteria elicit the production of antibodies by the immune system, which can also explain high population prevalence of mycoplasma. Vultures are exposed to many pathogens because they consume carcasses and roost communally, therefore they have strong immune systems [95,98]. Strong immune systems can establish robust defence mechanisms and provide protection against mycoplasma infections. Thus, it is possible that immunity to mycoplasma is high in vultures, allowing even a pathogenic bacteria to be prevalent in the population while exhibiting only low levels of pathology. Indeed, mycoplasma prevalence is generally very high in other raptor species; for instance, it reaches 91% in nest sites of *Circus aeroginosus* and *Milvus milvus*, as well as 94% in adult birds [28]. Additionally, in griffon vultures, the prevalence of mycoplasma was recorded at 47% and 70% in previous studies by Blass *et al*. [101] and Anglister *et al*. [29], respectively. Finally, some of the high prevalence values in our study come from sampling days on which sample sizes are low—for example with two out of two sampled individuals being positive (table 1), which is often a challenge in studies of infectious diseases in wildlife [102]. Still, on the sampling dates when we had larger sample sizes, we also observed high prevalence (table 1), indicating that small sample sizes are not the main driver for the high observed prevalence. Furthermore, removing the days with the low sample sizes and high prevalence from the analysis did not impact our results (electronic supplementary material, table S7). Further investigations into the causes underlying the high prevalence of mycoplasma bacteria in griffon vultures might provide important information on whether there is need to manage its spread and what such management might entail.

When examining infection with bacteria at the genus level it is not always possible to determine transmission directly, because infected individuals might be carrying different species of the bacteria. Indeed, multiple *Mycoplasma* species have been identified in this population and they differ in their origin (e.g. some arrive with translocated individuals from the Iberian peninsula [29]). It is further possible that transmission dynamics differ among bacteria species. Our analysis focused on the *Mycoplasma* genus because the prevalence at each identified species was too low to allow for separate analyses of social interactions [29]. Future work on transmission dynamics of mycoplasma in this system should focus on specific species or strains of the bacteria, and on describing how long vultures take to clear an infection [103] and whether they can become reinfected with the same, or with a different, mycoplasma strain using repeated samples of individuals.

Previous analyses showed that age is related to mycoplasma prevalence in griffon vultures, with immature individuals having higher mycoplasma than adults [29]. However, our analysis did not reveal such an effect of age. This difference between the two studies that examine the same population of griffon vultures can be explained by the difference in sample sizes. Anglister *et al*. [29] considered larger sample sizes, including samples of mycoplasma taken over a longer duration (2019–2022) of both captive and wild vultures, and included repeated samples of some individuals (*n* = 167 individuals and 244 mycoplasma samples). In our study, we considered a shorter period of mycoplasma sampling (2021–2022) because only this period had sufficient information about social behaviour. Furthermore, we included data only from wild individuals and considered a single bacterial sample (the first one taken) from each individual (*n* = 114 individuals, and 77 mycoplasma samples). Despite the smaller sample size in our study, immature individuals still tended to have higher (but not statistically significant) positivity than adults (table 2).

In conclusion, the social behaviour of wild griffons does not appear to influence mycoplasma infection. Identifying the reasons behind the high prevalence of mycoplasma in the population is crucial for guiding appropriate management strategies and protecting griffon vultures. Future use of theoretical models could help explore the potential dynamics of this bacteria to develop effective control strategies and mitigate its impact. Pathogens and infectious diseases have been identified as potential contributors to population declines and species extinction, and vaccination has been recommended to reduce the impact of infectious diseases on threatened wildlife populations [104–106]. Thus, it is essential to consider ecological and social contexts when examining disease prevalence due to their potential impact on disease spread in the population. While the social behaviours of hosts are often studied to understand the spread of pathogens, considering pathogen conditions is often neglected (e.g. commensals becoming pathogenic and pathogens causing chronic diseases). Understanding both host social interactions and pathogen biology is crucial for developing effective disease control strategies.

## Data Availability

Data are provided as part of the supplementary information [107], and analysis code is available on GitHub [108].
